# Precision Killing of M2 Macrophages with Phage-Displayed Peptide-Photosensitizer Conjugates

**DOI:** 10.3390/cancers15072009

**Published:** 2023-03-28

**Authors:** Mouldy Sioud, Qindong Zhang

**Affiliations:** 1 Department of Cancer Immunology, Division of Cancer Medicine, Oslo University Hospital, Radiumhospitalet, Ullernchausseen 70, 0379 Oslo, Norway; 2 Department of Pharmacy, Faculty of Mathematics and Natural Sciences, University of Oslo, Blindern, 0316 Oslo, Norway

**Keywords:** tumor microenvironment, macrophages, phage display, photosensitizers

## Abstract

**Simple Summary:**

Tumor-associated M2 macrophages impair the anti-tumor immune response and negatively impact clinical outcomes. Hence, a strategy that eradicates these immunosuppressive cells could hold great therapeutic potential. Here, we demonstrated that phage-displayed peptides conjugated to the IR700 photosensitizer can kill M2 macrophages after near-infrared light irradiation, while leaving M1 macrophages unaffected. Additionally, combining IR700 with a phage displaying a cancer-specific peptide killed both cancer cells and M2 macrophages. The finding that the wild type M13 phage has some tropism to M2 macrophages would expand the scope for phage-based therapeutics.

**Abstract:**

Among the immunosuppressive cells recruited to the tumor microenvironment, macrophages are particularly abundant and involved in angiogenesis, metastasis, and resistance to current cancer therapies. A strategy that simultaneously targets tumor cells and macrophages, particularly pro-tumoral M2 macrophages, would have significant clinical impact for various types of solid malignancies. By the use of phage display technology, we have recently developed a synthetic peptide, named NW, which binds to M1 and M2 macrophages with high affinity. Additional affinity selection on M2 macrophages identified only dominant peptides whose binding motifs are similar to that of the NW peptide. To reduce the frequency of selecting such dominating peptides, the peptide library was affinity selected on M2 macrophages blocked with NW peptide. This approach resulted in the selection of peptides that bind to M2, but not M1 macrophages. To explore the therapeutic potential of the selected peptides, the M13 phage-displayed peptides were conjugated to the photosensitizer IR700, which has been used for cancer photoimmunotherapy. The phage displaying a dominant peptide (SPILWLNAPPWA) killed both M1 and M2 macrophages, while those displaying the M2-specific peptides killed M2 macrophages only upon near-infrared light exposure. A significant fraction of the M2 macrophages were also killed with the untargeted M13 phage-IR700 conjugates. Hence, M2 macrophages can also be selectively targeted by the wild type M13 phage, which displayed a significant tropism to these cells. The benefits of this photoimmunotherapy include an automatic self-targeting ability of the wild type M13 phage, and the option of genetic manipulation of the phage genome to include tumor targeting peptides, allowing the killing of both M2 macrophages and cancer cells.

## 1. Introduction

Cancer immunotherapy is a concept based on the use of the -patients’ own immune system to fight off cancer [1]. Several therapeutic options have been explored to enhance treatment potency, including the use of engineered T cells and blocking antibodies directed against suppressive receptors [1]. However, despite the promise of these forms of immunotherapy, only a small fraction of treated patients respond well to treatment and yet fewer achieve a durable response [2]. The problem is that tumors possess an inherent ability to survive and use a diverse range of mechanisms to avoid killing by T cells. More importantly, T cells must overcome an often immunosuppressive microenvironment to recognize and kill tumor cells [3,4,5,6]. Among myeloid cells, tumor-associated macrophages (TAMs) are the most abundant immune cells infiltrating the tumor microenvironment (TME) [7,8,9,10]. While macrophages have central functions in tissue homeostasis and immunity, they have also been linked to numerous pathological processes including cancer, allergic inflammation and rheumatic inflammatory diseases [11,12].

Macrophage polarization is usually characterized by two states, namely M1 and M2 [11]. M1 macrophages are considered proinflammatory when activated, meaning that they play a central role during host defense. However, this also indicates that uncontrolled activation of M1 macrophages can lead to chronic inflammation. On the other hand, M2 macrophages play the opposite role, by decreasing inflammation and promoting tissue repair [12]. Notably, the polarization to M1 and M2 phenotypes is reversible, and macrophages can switch between the two depending on different activation signals [11]. With respect to cancer, M2 macrophages promote important features of tumor progression, including vascularization, cell proliferation, disease progression, and resistance to treatment, leading to poor disease outcome [2]. Given these observations, targeting of M2 macrophages represents a promising strategy for cancer immunotherapy. To therapeutically target TAMs, both immunological and pharmacological approaches have been explored [8]. For example, blocking the binding of colony-stimulating factor 1 (CSF-1) to its receptor (CSF-1R) expressed on monocytes/macrophages with an anti-CSF-1R antibody, Emactuzumab, reduced the number of infiltrated TAMs and increased the CD8+/CD4+ T cell ratio in mouse models for cancer [13]. CSF-1 and its CSF-1R regulate the migration, differentiation, and survival of macrophages [14]. Emactuzumab was used to treat patients with diffuse type giant cell tumors, resulting in clinical improvements that correlated with a reduction in TAMs and circulating monocytes [13]. Similarly, inhibition of CSF-1 gene expression with antisense oligonucleotides or small interfering RNAs, suppressed tumor growth in mice xenografted with human cancer cells [15,16]. The treatment also resulted in a significant reduction in macrophages in tumor tissues as compared to control animals. Depletion of macrophages in tumors has also been achieved by certain chemical drugs, e.g., trabectedin and bisphosphonates [17,18,19]. For instance, in biopsy samples obtained from sarcoma patients treated with trabectedin, the number of TAMs was significantly reduced [17]. Although these results are encouraging, the used methods deplete all macrophage subsets, including M1 macrophages, which have anti-tumoral functions. Hence, strategies for targeting specific TAM subsets are warranted.

By the use of peptide phage libraries combined with extensive screening and counter screening on non-target cells, we were able to select peptides that display binding to myeloid cells such as M1 and M2 macrophages [20,21]. One of the selected peptides, named NW, was conjugated to lytic peptides that killed both M1 and M2 macrophages [22]. The receptor of the NW peptide was recently identified as prohibitin-1 expressed on the surface of monocytes, M1 and M2 macrophages [23]. In this study, we present additional peptides targeting prohibitin-1, as well as new surface protein(s), found in the peptide library. Additionally, the cytotoxicity of the phage-displayed peptides when conjugated to the photosensitizer IR700 was evaluated using M1 and M2 macrophages. The results of these experiments are encouraging with respect to the ability -to selectively target and kill M2, while leaving normal anti-tumoral M1 macrophages unaffected.

## 2. Materials and Methods

### 2.1. Monocyte Purification from PBMCs

Monocytes were isolated from peripheral blood mononuclear cells (PBMCs) using plastic adherence, as previously described [22]. Briefly, PBMCs were purified from healthy donor buffy coats by density gradient centrifugation using Lymphoprep™ (Stemcell™ technology, Vancouver, BC, USA, Cat#07851) according to the manufacturer’s instructions. The isolated PBMCs were washed twice in PBS supplemented with 2% fetal bovine serum (FBS) and resuspended in complete RPMI 1640 medium. The cells were then seeded in T75 culture flasks (~50 million cells/flask) and allowed to adhere to the plastic surface of the culture flasks at 37 °C in a humidified incubator supplemented with 5% CO_2_ for 2 h. Adherent cells were collected by gentle scraping and resuspended in complete RPMI 1640 medium. Healthy donor buffy coats were obtained from the blood bank at Ullevål hospital, Oslo, Norway (project code: F8).

### 2.2. Generation of M1 and M2 Macrophages

To generate M1 macrophages, isolated monocytes were cultured in serum free X-vivo 15 medium (Lonza, Basel, Switzerland, Cat#BE02-060Q) supplemented with antibiotics, whereas complete RPMI medium was used for M2 macrophages. M1 and M2 macrophages were firstly primed by 50 ng/mL GM-CSF (Biotechne, R&D systems, Santa Clara, CA, USA, Cat#215-GM-010) and 50 ng/mL M-CSF (Biotechne, R&D systems, Cat#216-MC-035) for 4–6 days, respectively. Thereafter, the cells were cultured for additional two days in the presence of 50 ng/mL LPS and 100 U/mL IFN-γ (M1) or 50 ng/mL IL-4 (Biotechne, R&D systems, Cat#204-IL/CF) and 1 ng/mL IL-10 (Biotechne, R&D systems, Cat#217-IL) (M2). Under these conditions, the cells displayed the morphological characteristics of M1 or M2 macrophages [22].

### 2.3. Biopanning of Phage Peptide Library on M2 Macrophages

The 12-mer peptide phage library (Ph.D.™-12) was purchased from New England BioLabs (Ipswich, MA, USA). The phage library was amplified and titered according to the manufacturer’s instructions. The M1 and M2 macrophages used for biopanning were grown in T25 flasks until they reached 80–90% confluency on the day of the experiment. Prior to biopanning, the cells were washed in PBS buffer supplemented with 5% bovine serum albumin (BSA) (washing buffer) to remove dead cells. Thereafter, the phage library (10^11^ transduction units, TU), diluted in 3 mL washing buffer, was added to the cells and incubated for 1 h at room temperature with gentle agitation. Subsequently, the supernatant was removed and 10 mL washing buffer was added to the cells. The cells were then harvested by gentle scraping and transferred to a 15 mL Falcon tube. The cells were pelleted by centrifugation at 300× *g* for 3 min and then washed 10 times with the washing buffer to remove unbound phages. Cell-binding phages were eluted in 200 μL elution buffer (0.1 M glycine-HCl, pH2.2, 0.1% BSA) for 10 min at RT with constant rotation followed by centrifugation for 5 min at 12,000× *g*. The supernatant containing the eluted phages was collected and neutralized with 28 μL neutralization buffer (Tris-HCl, pH 9.2). The eluted phages were amplified in the *E. coli* ER2738 strain, titered and then used in subsequent rounds of biopanning. In addition, the biopanning was performed with subtraction steps, in which the phage library was pre-incubated with peripheral blood mononuclear cells and/or M1 macrophages prior to affinity selection on M2 macrophages. This pre-incubation step was carried out from round 1. After 3 rounds of biopanning, the enrichment of specific phages was determined by flow cytometry, followed by isolation and sequencing of single phages. For the NW peptide blocking strategy, the M2 macrophages were incubated with 200 μg of the NW peptide for 1 h at RT prior to biopanning as described above. Three rounds of biopanning were performed on blocked M2 macrophages. Similarly, the library was pre-incubated with PBMCs prior to affinity selection on blocked M2 macrophages.

### 2.4. Analysis of Phage Binding to Macrophages by Flow Cytometry

Briefly, aliquots of M1 and M2 macrophages containing 1–2 × 10^5^ cells were dispersed to conical 96-well V-bottom microplates and then incubated with the amplified phages (10^9^ TU/mL) for 30–45 min on ice. After washing with PBS buffer containing 3% FBS, cells were incubated with biotinylated anti-M13 monoclonal antibodies (Nordic BioSite, Täby, Sweden, Cat#158-11973-MM05T-B-100) for another 30 min on ice. Thereafter, washed cells were stained with phycoerythrin (PE)-conjugated streptavidin for 30 min on ice in darkness. Competition assays were performed by pre-incubating M2 macrophages with different amounts of NW peptide or a mutant form of NW peptide for 20 min at RT before adding the phage-displayed peptides. The samples were incubated for 40 min on ice, washed, and incubated with PE-conjugated streptavidin prior to flow cytometric analysis. In some experiments, secondary staining was performed with FITC- (Santa Cruz Biotechnology, INC, Cat#sc-53004 FITC, Dallas, TX, USA) or PE-conjugated anti-M13 monoclonal antibodies (Santa Cruz Biotechnology, INC, Cat#sc-53004 PE, Dallas, TX, USA). All secondary staining involved 30 min of incubation at 4 °C in darkness. After washing, the cells were analyzed on a BD FACS Canto II equipped with the BD FACS DIVA software (BD Biosciences, San Jose, CA, USA). Data analysis was performed with FlowJo version 7.6.5 (FlowJo LLC, Ashland, OR, USA).

### 2.5. Analysis of the Phage-IR700 Binding to Macrophages

Flow cytometry was used to investigate the binding of the phage-IR700 conjugates to M1 and M2 macrophages. Cells were incubated with the phage particles (10^7^ TU/mL) in staining buffer (3% BSA in PBS) for 40 min at 4 °C. After washing with staining buffer, the samples were analyzed by flow cytometry using a Cytoflex S cytometer (Beckman Coulter Life Sciences, Indianapolis, IN, USA) equipped with the Cytexpert 2.1 software (Beckman Coulter). Results were analyzed by FlowJo version 7.6.1 (FlowJo LCC, Ashland, OR, USA).

### 2.6. Conjugation of IR700 to Phage-Displayed Peptides

Phage particles (10^10^ TU) were incubated with IR700DX NHS ester (20 μg) in 1 mL 100 mM Na_2_HPO_4_, pH 8.5 at RT for 1 h with gentle rotation. Subsequently, the reaction was quenched by the addition of 100 μL 250 mM glycine buffer (pH 7.5). Phage particles were precipitated by mixing a 1:5 volume of 20% polyethylene glycol (PEG) 8000/2.5 M NaCl with the reaction sample, followed by incubation at 4 °C for 2 h. The samples were spun down at 12,000× *g* for 15 min to recover the phage pellets. PEG precipitation was repeated twice. After the first precipitation, each phage pellet was resuspended in 1 mL PBS buffer, vortexed for 30 s and then PEG precipitated again. Thereafter, the phage pellets were dissolved in 150 μL PBS and stored at 4 °C until use. Under our experimental conditions, each phage particle bound on average 2 to 4 × 10^3^ IR700 molecules deduced from a standard calibration curve that was constructed using a set of standard samples of known IR700 concentrations. UV-Vis absorption spectra were acquired on a Shimadzu spectrophotometer (UV2550) in 1 mL quartz cell at room temperature in PBS buffer. The spectra were carried out between 200 nm and 900 nm, then values were collected and processed (Supplemental Figures S1 and S5). The samples were also analyzed by SDS-PAGE under denaturing conditions (Supplemental Figures S3 and S4).

### 2.7. DNA Sequencing

DNA from individual positive phage clones were isolated using single-stranded M13 DNA isolation kit (Qiagen Norge, Oslo, Norway). The sequences of the phage-displayed peptides were deduced by sequencing the unique nucleotide region of the pIII protein using a specific primer (Eurofins MWG, Ebersberg, Germany).

### 2.8. Photocytotoxicity

Blood monocytes (2 × 10^4^ cells/well/100 μL) were first differentiated into M1 and M2 macrophages in 96-well flat-bottom plates to generate 70–80% confluent cells on the day of the experiments. On day 7 post monocytes differentiation, dead cells were removed by replacing the medium. Then, the cells were incubated with the test molecules for 1 h at room temperature. After washing with serum free medium to remove unbound phages, 100 μL complete medium was added to each well and the cells were exposed to near infrared light (30 J/cm^2^). For this purpose, an in-house built lamp comprising an array of 24 light-emitting diodes (LED, 690 nm) was used. The power density of the LED array at the surface of culture plate was 17 mW/cm^2^. After light irradiation, the cells were placed in the incubator (dark) for various time periods and cytotoxicity was measured using the CellTiter 96 AQueous One Solution Cell Proliferation Assay according to the manufacturer’s instructions (Promega, Madison, WI, USA). Optical densities were measured at 492 nm using a 96-well plate reader (TECAN, Sunrise; Männedorf, Switzerland). Plates that were not NIR-irradiated were shielded from ambient light. In some experiments, fluorescein diacetate (FDA) was used in combination with propidium iodide (PI) to stain live and dead cells, respectively.

### 2.9. Bright-Field and Fluorescence Imaging

An inverted Zeiss Axiovert 40CFL microscope (Carl Zeiss AG, Jena, Germany) was used to image cells exposed to various treatments. Cells growing in 96-well plates were imaged with a 40× objective (LD A-Plan 40×/0.50 Ph2 Zeiss). For fluorescence images, excitation light from a 50 W super-pressure mercury lamp was used together with appropriate beam splitters and filter combinations. For red fluorescence (PI), a beam splitter (filter set number 00, Carl Zeiss) with an excitation bandpass filter of 530–585 nm and emission longpass filter of 615 nm were used. For green fluorescence (FDA), a beam splitter (filter set number 09, Carl Zeiss) with an excitation bandpass filter of 450–490 nm and emission longpass filter of 515 nm were used. All data were acquired and analyzed using the Carl Zeiss AxioVision software, version 4.8.2.

### 2.10. Docking

The molecular docking between prohibitin-1 and the different peptides was conducted using HEPDOCK, a server for blind prediction of peptide-protein docking based on a hierarchical algorithm [24], http://huanglab.phys.hust.edu.cn/hpepdock/ (accessed on 15 August 2022). The structural data for prohibitin-1 (PDB ID:6IQE) is available from the RCSB PDB databank.

### 2.11. Statistical Analysis

Data are expressed as mean ± SD based on minimum of three experiments, unless otherwise indicated. Statistical analysis was carried out with GraphPad Prism version 4 (GraphPad Software, Inc., La Jolla, CA, USA). Statistical significance was evaluated by Student’s *t*-test or non-parametric ANOVA test.

## 3. Results

### 3.1. Biopanning on M2 Macrophages Reveals the Importance of Prohibitin-1 in Phage Selection

**Figure 1:**
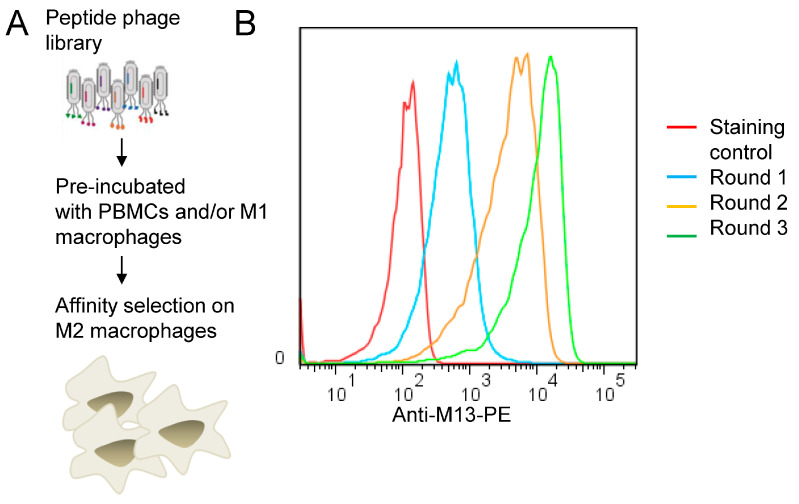
(**A**) Biopanning on M2 macrophages. Schematic representation of the biopanning protocol. The peptide phage library was pre-incubated with non-target cells and then affinity selected on M2 macrophages. (**B**) Representative flow cytometry histograms showing phage pool binding from round 1, 2 and 3 to M2 macrophages.

**Figure 2:**
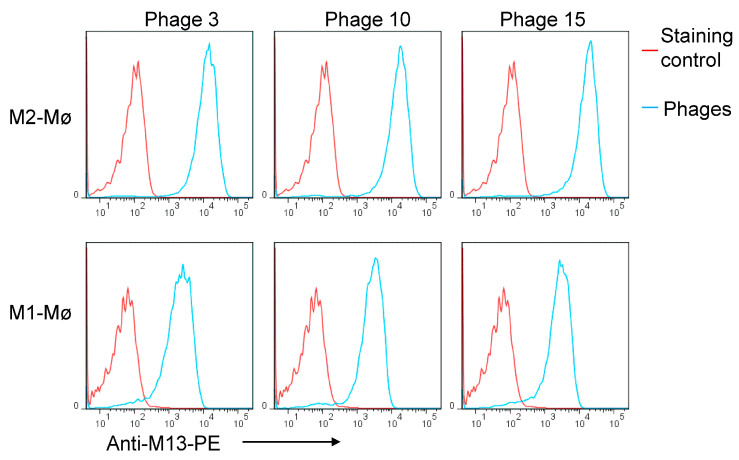
Binding of single phage clones to M1 and M2 macrophages. Representative examples of flow cytometry histograms showing the binding of single phage clones from round 3 to M1 and M2 macrophages. All tested phage clones bound to M1 and M2 macrophages with high affinity.

**Figure 3:**
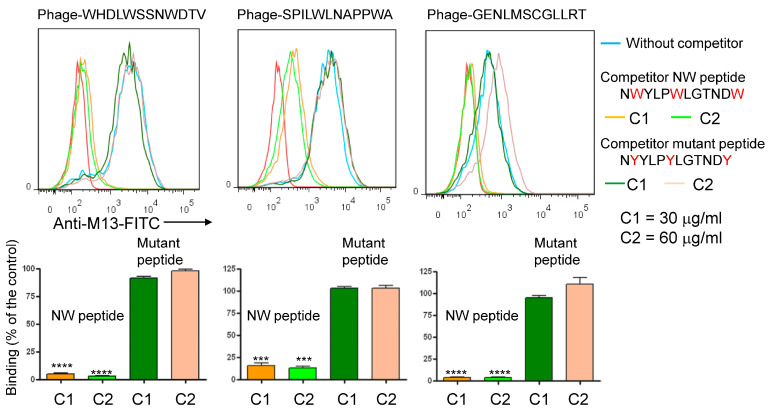
Inhibition of the phage-displayed peptide binding by NW peptide. M2 macrophages were pre-incubated with either the NW peptide or its mutant form and then stained with the indicated phage-displayed peptides (10^7^ TU/mL) followed by flow cytometry analysis. The binding of the phages was detected using FITC-conjugated anti-M13 monoclonal antibody. Lower panels represent the percentage of inhibition. Florescence of cells without competitor is taken as a control (100%). *** *p* < 0.001, **** *p* < 0.0001.

**Figure 4:**
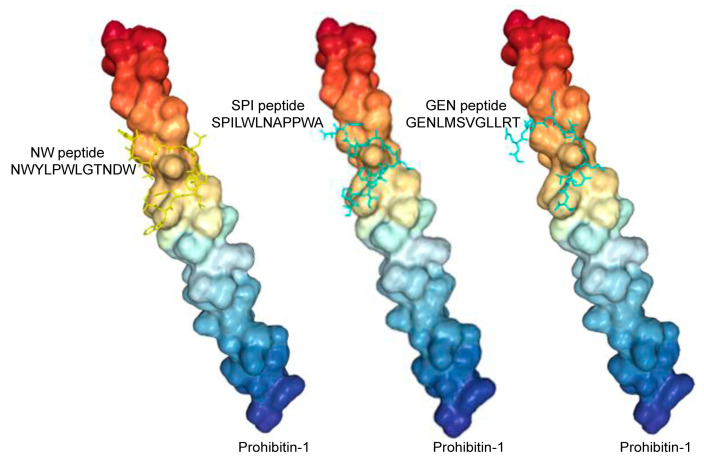
Representative docking results showing the predicted prohibitin-1 binding sites for the peptides. Several of the high affinity peptide predicted 3D structures bind to the same area. Protein style = surface; peptide style = licorice.

In the context of cancer, high macrophage infiltration is associated with poor prognosis in most human solid tumors. Most, if not all, TAMs have the pro-tumoral M2 phenotype and are mainly replenished by the circulating myeloid precursor pool [9]. To develop a targeting strategy using short peptides that recognize cell surface receptors expressed on M2 macrophages, we have screened a random peptide phage library on these cells. To enrich for specific binders, the phage library was pre-incubated on healthy donor peripheral blood mononuclear cells (PBMCs) and M1 macrophages to exclude phages that bind to common cell surface receptors. Then, the pre-absorbed 12-mer random peptide library was biopanned on M2 macrophages as illustrated in Figure 1A. Subsequent to the third round of selection, polyclonal phages were amplified and tested by flow cytometry. Enrichment of positive phages was evident after the 2nd and 3rd rounds, indicating successful selection of positive phage clones (Figure 1B). The biopanning strategy was repeated several times using M2 macrophages derived from different donors, with or without the subtraction step. Subsequently, single phages were isolated and tested by flow cytometry. Most of the selected phages displayed strong binding to both M1 and M2 macrophages (Figure 2, as representative examples). Sequence analysis of the selected phages from four independent biopanning experiments revealed that the peptide sequences of all positive phages were similar to a peptide named NW (NWYLPWLGTNDW) which we have previously identified [21] (Table 1). Competition experiments revealed that the selected peptides bound to the same receptor as the NW peptide, namely prohibitin-1 expressed on the surface of monocytes, macrophages and dendritic cells (Figure 3, as representative examples). In contract to the mutant peptide, the NW peptide inhibited the phage binding to M2 macrophages. Additionally, most of the predicted peptide structures dock at the same binding site as the NW peptide (Figure 4, as representative examples). The results presented above indicated that prohibitin-1 is an important driver in the selection process. Moreover, the subtraction step using M1 macrophages and/or PBMCs was unsuccessful since the selected clones bind to both M1 and M2 macrophages, as well as blood monocytes. Additionally, high affinity peptides such as SPI (SPILWLNAPPWA) and WHD (WHDLWSSNWDTV) were selected whether or not the subtraction step was included. Interestingly, a low affinity peptide (GENLMSVGLLRT) mimicking the NW structure was also selected. Indeed, the binding of the phage displaying this peptide was inhibited by the NW peptide, but not the control peptide (Figure 3, right panel).

### 3.2. Blocking with NW Peptide Results in the Enrichment of M2-Binding Phages

**Figure 5:**
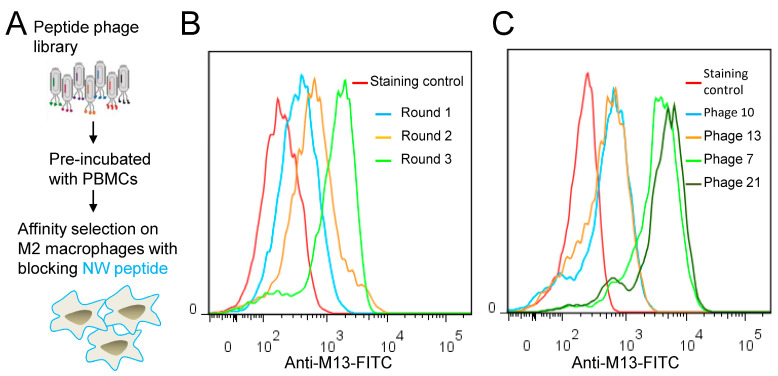
Selection on target cells with blocking NW peptide. (**A**) Schematic illustration of peptide blocking strategy. The peptide phage library was pre-incubated with non-target cells and then affinity selected on M2 macrophages with NW peptide blocking. (**B**) Binding of the enriched phage pool from round 1, 2 and 3 to M2 macrophages. (**C**) Representative examples of binding patterns of single phage clones to M2 macrophages. Cells were incubated with single phage clones (10^7^ TU/mL) followed by FITC-conjugated anti-M13 monoclonal antibody staining and analysis by flow cytometry.

To prevent re-selection of prohibitin-binding phages and facilitate the isolation of new peptides, we hypothesized that blocking the cells with NW peptide during biopanning would reduce the fraction of phages displaying similar peptides in the output pool (Figure 5A). After three rounds of selection, amplified polyclonal phages were tested by flow cytometry (Figure 5B). The specificity of the phage pools from the first, second, and third round of selection was analyzed for binding to M2 macrophages. When comparing the fluorescent signal from each round of selection, there was evident enrichment of positive clones after the third round. Thereafter, single phage clones were amplified and tested by flow cytometry. Under our experimental conditions, two categories of phages were selected; namely high and low affinity binders (Figure 5C).

**Figure 6:**
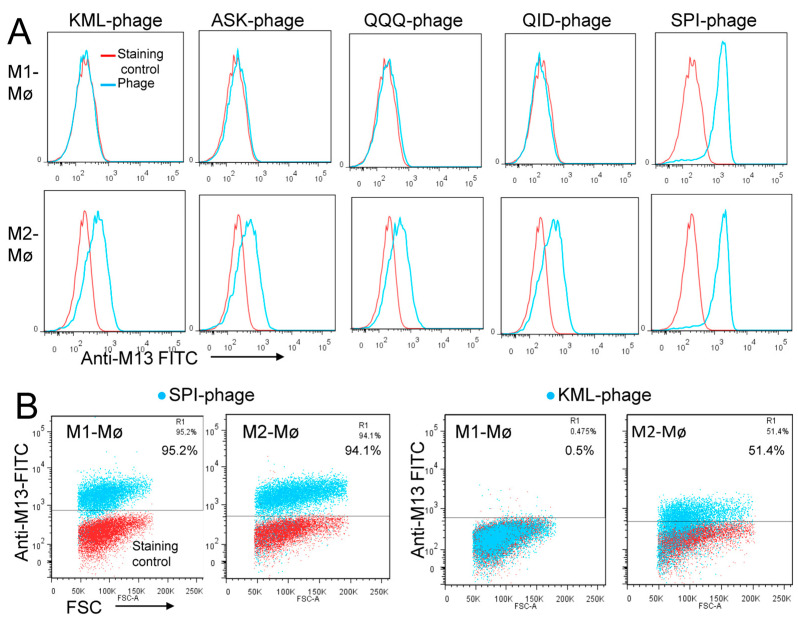
Flow cytometry analysis of the phage binding. (**A**) Representative examples of flow cytometry histograms showing the binding of low affinity phage-displayed peptides to M1 and M2 macrophages. The phage displaying SPI peptide (SPI phage) was included as a positive control that binds to both macrophage subsets. Binding was detected using FITC conjugated an anti-M13 monoclonal antibody. (**B**) To better visualize the percentage of positive cells, the binding of the phage displaying KML peptide (low affinity) and SPI peptide (high affinity) were also presented as dot plots.

To evaluate the specificity of the clones enriched from enriched from the peptide blocking experiments, 30 phage clones were isolated and sequenced. All the high affinity peptides displayed the sequence TWDLPWLLEKPF, which is similar to the NW peptide (Table 2). Six sequences were identified among the low affinity phage clones and certain peptides shared consensus sequences, suggesting that their selection was receptor-driven. The low affinity binders bound to M2, but not to M1 macrophages (Figure 6A,B). Moreover, the NW peptide did not compete with the binding of the low affinity peptides to M2 macrophages, indicating that their receptors are different from prohibitin-1 [25]. Overall, the removal of the most abundant clones through blocking with NW peptide resulted in the selection of low affinity peptides with specificity for M2 macrophages, which can be used for specific targeting of these immunosuppressive myeloid cells.

### 3.3. Photocytotoxicity of the Phage Displayed Peptide-Photosensitizer Conjugates

**Figure 7:**
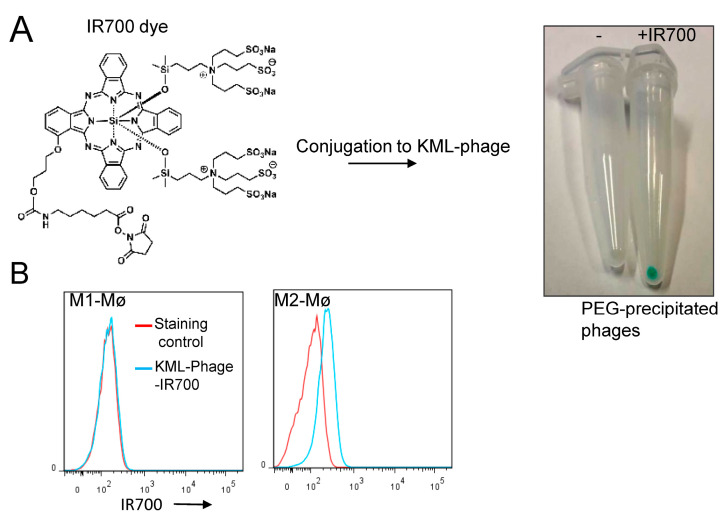
Conjugation of the phages to IR700 photosensitizer. (**A**) Chemical structure of the IR700 photosensitizer. After PEG precipitation, a picture was taken to illustrate the conjugation of the IR700 dye to the phage particles. (**B**) Flow cytometry analysis of the conjugate binding to M1 and M2 macrophages. The cells were -incubated with the conjugate (10^7^ TU/mL) and then analyzed by flow cytometry.

The selection of M2 macrophage-specific phage clones should pave the way for multiple targeted therapies. Recently, we have developed an improved version of photoimmunotherapy that uses single chain Fv antibodies to direct the IR700 photosensitizer to cancer cells [26]. Given its high hydrophilicity, the IR700 photosensitizer, developed by Kobayashi’s lab, requires a ligand to bind and kill target cells after near infrared (NIR) light exposure [26,27,28]. Hence, we hypothesized that the combination of IR700 with the phage displaying the KML peptide (M2-specific) may kill M2, but not M1 macrophages. We first conjugated the IR700 to the phage and then investigated the binding of the conjugate to macrophages. To purify the conjugate, we took advantage of the interaction of phages with polyethylene glycol (PEG), a frequently used method to purify phage populations for downstream applications, as illustrated in Figure 7A [29]. Purification resulted in a high yield of the conjugated phage in a correctly folded form. Indeed, all phage-IR700 conjugates were able to infect the ER2738 *E. coli* strain and exhibited similar titer to unmodified phages. Additionally, the conjugate bound to M2 macrophages (Figure 7B, as a representative example). Under our experimental conditions free non-conjugated IR700 molecules were not recovered after PEG precipitation (Supplemental Figures S1, S2 and S5). Collectively, these results indicate that the phage is able to maintain its structure and binding to M2 macrophages when in conjunction with the IR700 photosensitizer.

**Figure 8:**
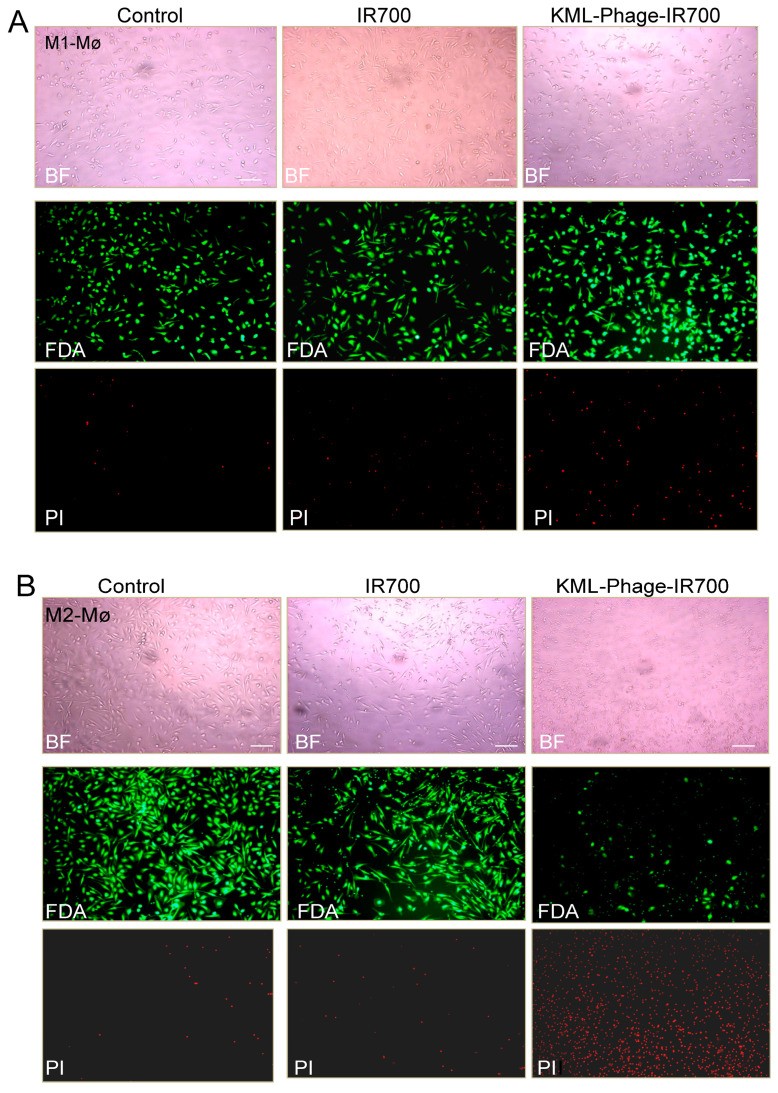
Photocytotoxicity of the KML Phage-IR700 conjugate. M1 (**A**) and M2 (**B**) macrophages were incubated with either IR700 dye (50 nM) or the KML-phage-IR700 conjugate for 1 h at room temperature, washed, and then exposed to NIR light 35 J/cm^2^ at power density of 17 mW/cm^2^. Thereafter-, the cells were incubated at 37 °C for 3 h and then Light fluorescence microscopy images of cells counter-stained with fluorescein diacetate (FDA) and propidium iodide (PI) were taken. Scale bars present 100 μm. BF, bright field.

Having demonstrated the binding to M2 macrophages, we proceeded investigating the photocytotoxicity of the KML phage-IR700 conjugate. Our preliminary experiments indicated that a concentration of 10^7^ TU/mL of the phage, which contains on average 2 to 4 × 10^3^ IR700 molecules per phage, is effective in killing M2 macrophages upon near-infrared (NIR) light exposure (35 J/cm^2^). First, the viability of M2 and M1 macrophages was determined using fluorescein diacetate (FDA) and propidium iodide (PI) double staining. Cells growing in 96-well plates were incubated with the KML-phage- IR700 conjugate or IR700 dye for one hour at room temperature, washed to remove the unbound phage particles, and then exposed to NIR light (35 J/cm^2^). After incubation for 3 h at 37 °C the cells were stained with FDA and PI. Live cells should convert the non-fluorescent FDA into the green fluorescent compound fluorescein, while compromised cells will emit red light, a sign of cell death. In contrast to M1 macrophages (Figure 8A), all M2 macrophages treated with the KML phage-IR700 conjugate were killed, while those untreated and treated with IR700 dye only remained viable (Figure 8B). Light microscopy also revealed severe alterations in the cell morphology post-NIR irradiation (Upper panels). We and others have shown that once the monoclonal antibody-IR700 conjugate binds to the target cell and is exposed to NIR light, it can result in rapid and irreversible damage to the cell membrane [26,28,30].

**Figure 9:**
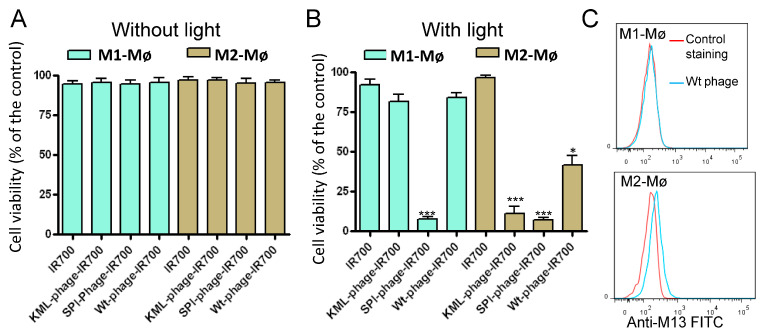
Analysis of the phage-IR700 conjugate photocytotoxicity by MTS assay. M1 and M2 macrophages were incubated with IR700 (50 nM) or phage-IR700 conjugates (10^7^ TU/mL, with approximately 2250 IR700 molecules/phage) at room temperature for one hour. After washing, the cells were not (**A**) or exposed (**B**) to light (35 J/cm^2^ at power density of 17 mW/cm^2^) and then incubated at 37 °C for 16 h. After, cell viability was measured by MTS assay. As controls, the phage displaying the SPI peptide (SPI-phage-IR700) and wild phage (Wt-phage-IR700) were included. Data are expressed as percentage of the control cells (untreated) and are from triplicate determinations. Data are representative of 3 independent experiments. * *p* < 0.03, *** *p* < 0.001. (**C**) Analysis of the wild type (Wt) phage interaction with M2 macrophages. The cells were incubated with the Wt phage (10^7^ TU/mL) and then analyzed by flow cytometry. Binding was detected using FITC conjugated an anti-M13 monoclonal antibody.

We next quantitatively evaluated the cell viability using the Promega CellTiter 96™ AQueous One Solution, which contains an MTS agent that is converted by mitochondrial enzymes of living cells to form formazan. For comparison, we included the phage displaying the SPI peptide, which binds to both M1 and M2 macrophages with high affinity (see Figure 6A,B). In addition, we also included the untargeted wild type M13 phage (Wt-phage). By contrast to non-conjugated phages, the conjugated phages showed absorption at 689 nm that is specific for the IR700 dye (Supplementary Figure S1). Additionally, the major coat protein pVIII derived from IR700-conjugated phages migrated slower in SDS-PAGE than that derived from non-conjugated phages, supporting the covalent linkage between the protein and the dye (Supplementary Figure S4). M1 and M2 macrophages were incubated with the IR700 conjugated phage-displayed peptides for 60 min at room temperature, washed and then irradiated or not with NIR light (35 J/cm^2^). Cell viability was assessed around 16 h post-NIR irradiation. Cells incubated with the tested conjugates did not exhibit significant cell death (5% ± 2%) without light irradiation (Figure 9A) whereas NIR-irradiation of cells treated with the KML-phage killed 93% ±5% of M2 macrophages (Figure 9B, *p* < 0.001). As expected, the phage displaying the SPI peptide conjugated to IR700 killed both M1 and M2 macrophages. Surprisingly, a significant fraction of M2 macrophages were also killed with the untargeted M13 phage-IR700 conjugate (*p* < 0.03). The M13 phage seems to selectively adhere to M2 macrophages when compared to M1, as verified by flow cytometry analysis (Figure 9C). Although this interaction is weak, it can still be used to kill M2 macrophages when conjugated to a powerful photosensitizer such as IR700. Overall, the results of these experiments are encouraging with respect to the ability to selectively target and kill M2 while leaving normal antitumoral M1 macrophages unaffected.

### 3.4. Co-Targeting Cancer Cells and M2 Macrophages

**Figure 10:**
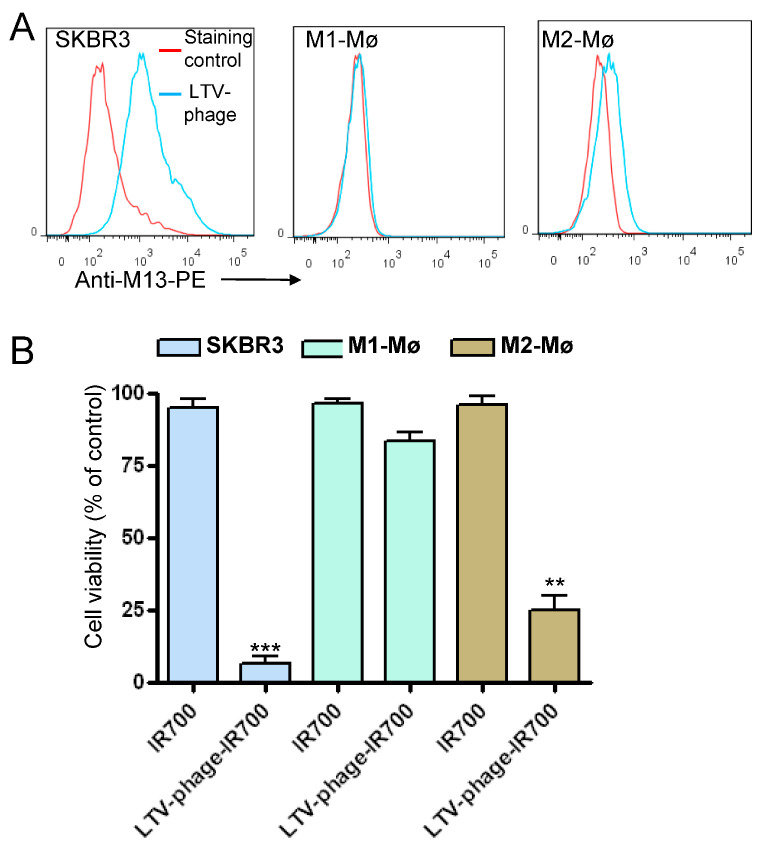
Dual targeting with the LTV phage-IR700 conjugate. (**A**) Representative histograms showing the binding of the phage displaying the LTV peptide to breast cancer cell line SKBR3, M1 and M2 macrophages. Cells were incubated with the phage particles (10^7^ TU/mL, with approximately 3800 IR700 molecules/phage) and then analyzed by flow cytometry. Binding was detected using PE conjugated anti-M13 monoclonal antibody. (**B**) Photocytotoxicity of the LTV-phage-IR700 conjugate on SKBR3 breast cancer cell line, M1 and M2 macrophages was measured by MTS assay. Experimental conditions are as in Figure 9. ** *p* < 0.02, *** *p* < 0.001.

Given the inherent affinity towards M2 macrophages, we investigated whether an M13 phage displaying a cancer-specific peptide could be used to kill both cancer cells and M2 macrophages. Previously, we have applied phage display to isolate multiple peptides with high affinity and specificity for a variety of cancer cells [20,31]. For example, the LTV-peptide that preferentially binds HER-2 positive cancer cells and has been used to guide the delivery of therapeutics and imaging agents to various tumor cell types [31,32,33,34,35]. As such, this peptide is an attractive vehicle to deliver photosensitizers to cancer cells. As shown in Figure 10A, the phage-displaying the LTV peptide demonstrated strong binding to SKBR3 cells. Similar to the wild type M13 phage, a certain level of binding to M2 macrophages was also detected (Figure 10A). The LTV phage conjugated to IR700 killed both SKBR3 and M2 macrophages after NIR light exposure (Figure 10B). Most importantly, the effect on M1 macrophages was minimal when compared to M2 macrophages. Untreated and cells treated with IR700 only were not affected by NIR light exposure. According to our concept, the engineered phage conjugate could represent an effective therapeutic approach to kill tumor cells and attenuate the immunosuppressive role of M2 macrophages in TME.

## 4. Discussion

Tumor-associated M2 macrophages are a critical component of the tumor microenvironment [12], where their tissue-regenerating phenotype is exploited to support tumor growth. Hence, considerable attention has been given to the development of cancer immunotherapies targeting M2 macrophages. However, in addition to inhibiting M2 macrophages, these therapies also deplete macrophages that play important roles in normal processes throughout the body, such as tissue remodeling and immune responses [11]. In this study, we showed that phage-displayed peptides targeting M2 macrophages can be selected and turned into effective photoimmunotherapy agents. For example, the phage-displayed KML peptide (KML-phage-IR700) was proven to target and selectively kill M2 macrophages upon NIR light irradiation. The finding that the wild type M13 phage can specifically interact with M2 macrophages should be further explored for directing photosensitizers to these immunosuppressive cells. As such, macrophage-directed therapeutic strategies have the potential to complement and synergize with current cancer therapies.

As discussed elsewhere, phage display is a technology that uses genetically modified phages to identify peptides and proteins with desired binding properties [20]. The screening process has proven to be an effective tool for the identification of peptides that can specifically bind to various target molecules [20,36]. While the target can be immobilized recombinant proteins, whole cell biopanning focuses on identifying peptides binding specifically to a single cell type. The biopanning cycle is normally repeated three to five rounds to ensure that only phages with high affinity to the target are enriched. One advantage of the phage library is that it allows a variety of depletion and competition steps to be performed to guide the selection. However, despite the pre-incubation of the library with non-target cells (e.g., blood monocytes, M1 macrophages), our initial attempts resulted in selection of phages that bind to both non-target and target cells. Additionally, only overrepresented peptides that bind to prohibitin-1 were selected. Despite being considered cytosolic proteins, prohibitins 1 and 2 have been identified on the cell surface of macrophages and other cell types [37,38]. While using two different 12-mer random peptide libraries and cells from different donors, the biopanning protocol on M2 macrophages led to the selection of only peptides similar to the NW peptide. The reason for this biased selection is not yet known.

To reduce the frequency of already discovered peptides, we applied a peptide blocking strategy where the phage library underwent affinity selection on whole cells in the presence of the NW peptide. Phage clones displaying peptides not previously observed were selected. Importantly, rare binders with M2 macrophage specificity were fished out from the phage pool. None of the so-called low affinity binders were selected using the standard selection protocols. These findings illustrate an approach to discover new peptides when biopanning is performed on complex targets such as whole cells. Some of the selected phage-displayed peptides were converted into phage-IR700 conjugates and shown to kill M1 and/or M2 macrophages. One of the unique characteristics of the developed phage-IR700 conjugate is that it gains photocytotoxicity only when it is bound to the target cell membrane and is activated by NIR light [26,27]. Only the phage-displayed peptides conjugated to the IR700 photosensitizer demonstrated cell killing when irradiated with NIR light (690 nm). Interestingly, we found that the weak interaction of the wild type M13 phage-IR700 conjugate with M2 macrophages is sufficient to kill these cells. As such, a specific M2 targeting peptide may not be needed for the present photoimmunotherapy. Accordingly, this implies the exciting potential of targeting both cancer cells and M2 macrophages using phages that display cancer-specific peptides, as demonstrated for the phage displaying the LTV peptide (Figure 10). Future studies will be necessary to determine how the M13 phage interacts with M2 macrophages, but not M1 macrophages, and if other bacteriophage members (e.g., fd, T4) display the same binding profile.

Notably, phages do not have inherent mammalian cell tropism, yet they can efficiently transcytose human tissues [39,40]. However, by expressing tumor targeting peptides on the surface of phages, thereby introducing such tropism, one could render the phage adaptable for cancer-targeted applications [20,36,41]. The specificity of the target is related to the proteins or peptides expressed on the phage surface, which can further be conjugated with other therapeutic agents, providing more specific targeting and drug-loading capacity. Depending on the targeted receptors, the recombinant phages can be internalized by endocytosis, thus opening the possibility to be used as delivery and/or imaging agents. For example, the bacteriophage MS2 was engineered to target Jurkat leukemia T cells by conjugating the phage capsid to photosensitizer porphyrins, while the outer capsid surface was chemically modified to bind Jurkat-specific aptamers [42]. Similarly, M13 phages displaying a peptide targeting the epidermal growth factor receptor were chemically modified with various photosensitizers [43,44]. The engineered conjugates selectively killed target cancer cells after light exposure. The use of the M13 phage as carrier for cancer cell-targeting peptides and photosensitizers was also reported by Gandra et al. [45]. Bacteriophages were used not only to deliver photosensitizers but also chemotherapeutic agents. In this respect, the antitumor drug doxorubicin was encapsulated into folate-conjugated phage nanoparticles and shown to navigate the drug to folate-receptor expressing cancer cells [46]. In another study, genetically engineered M13 phage nanoparticles were loaded with doxorubicin and tumor specificity was achieved by the display of the DKF motif [47]. DKF is recognized by cathepsin B, a lysosomal protease that is overexpressed in most prostate cancer cells. With well-defined molecular structures, phage nanocarriers offer unique opportunities for functional manipulation using genetic and/or chemical engineering. As demonstrated in this study, the IR700 photosensitizer can be used to turn the phage-displayed peptides into photoimmunotherapeutic agents with the potential to kill immune suppressive cells. As indicated above, M2 macrophages are associated with poor prognosis and resistance to current immunotherapies. They inhibit the anti-tumor immune responses through multiple mechanisms including the depletion of key amino acids (e.g., tryptophan, L-arginine), production of inhibitory factors (IL-10, TGF-β) that suppress T cell effector functions and recruitment of regulatory T cells to the TME [8,9]. Of note, tryptophan and L-arginine are essential for T cell function and survival. The development of strategies to navigate drugs such as photosensitizers to cancer cells should increase the selective accumulation of such agents in the tumor tissues and thus reduce undesirable side-effects [48,49,50,51,52,53,54]. The observation that the wild type M13 phage has some tropism to M2 macrophages will likely expand the scope for therapeutic phages. Notably, phages are known to be very common in the gastrointestinal tract and, together with their bacterial hosts, are an important component of gut flora [55,56]. Thus, all aspects of the interactions between phages and innate immune cells are of interest and importance for further medical and biochemical applications.

## 5. Conclusions

Our understanding of the role of immunosuppressive cells that reside in the TME has greatly increased over the past decade. Tumor-associated M2 macrophages impair the antitumor immune response and negatively impact clinical outcomes. Therefore, a strategy that kills these immunosuppressive cells could hold great therapeutic potential. Using a peptide blocking strategy, we demonstrated that phage-displayed peptides recognizing surface receptors expressed on M2 macrophages can be isolated from random peptide libraries. Depending on the displayed peptide, the conjugation of the phages with the IR700 photosensitizer killed M1 and/or M2 macrophages. The specific interaction, although weak, of the M13 phage with M2 macrophages should be further explored to deliver therapeutics to M2 macrophages. Overall, our findings lay a strong foundation for the future development of phage-IR700-based photoimmunotherapy targeting immune suppressive cells such as M2 macrophages, myeloid-derived suppressor cells and regulatory T cells.

## Figures and Tables

**Table 1:** Sequences of the phage-displayed peptides.

Selected Peptides	Binding	
	M1-Mø	M2-Mø
N**W**Y**LPWL**GTND**W** *	++++	++++
Q **W** E **LPWL** MQP **P** L	++++	++++
T **W** A **LPWL** LEK **PF**	++++	++++
SP **ILWL** NAP **PW** A	++++	++++
**W** HD **LW** SSN **W** DTV	++++	++++
GEN **L** MSVGLLRT	++	++

* Reference NW peptide. Common amino acids are indicated in bold. Binding of the phage clones to M1 and M2 macrophages was analyzed by flow cytometry. ++, binding < 50%; ++++, binding > 80–100%.

**Table 2:** Sequences of the phage-displayed peptides.

Selected Peptides	Binding	
	M1-Mø	M2-Mø
N**W**Y**LPWL**GTND**W** *	++++	++++
T **W** D **LPWL** LEKP **F**	++++	++++
KML **PTMPR** VLA **G**	-	++
DAA **PTLPK** GGV **G**	-	++
QID **TGY** GLVS **V** S	-	++
GSK **TGY** LSET **V** R	-	++
ASKNAHLFLSSL	-	+
QQQYGTYVPTFG	-	+

* Reference NW peptide. Common amino acids are indicated in bold. Binding of the phage clones to M1 and M2 macrophages was analyzed by flow cytometry. +, binding < 25%; ++, binding < 50%; ++++, binding > 80–100%. -, No significant binding.

## Data Availability

Public data sources are listed in methods.

## Supplementary Materials

The following supporting information can be downloaded at: https://www.mdpi.com/article/10.3390/cancers15072009/s1, Figure S1. UV-Vis absorbance spectra of the phage-IR700 conjugates after PEG precipitation; Figure S2. Polyethylene glycol (PEG) precipitation of the conjugated phages; Figure S3. Analysis of the IR700-conjugated phages by 15% SDS-PAGE; Figure S4. Analysis of non- conjugated and IR700 conjugate phages by 15% SDS-PAGE; Figure S5. UV-Vis absorbance spectrum.

## Abbreviations

CSF-1	Colony-stimulating factor 1
CSF-1R	Colony-stimulating factor 1 receptor
FDA	Fluorescein diacetate
IL	Interleukin
LED	Light-emitting diode
NIR	Near infrared
PBMC	Peripheral blood mononuclear cell
PE	Phycoerythrin
PEG	Polyethylene glycol
PI	Propidium iodide
TGF-β	Transforming growth factor β
Wt	Wild type
