# Supplementary material for: Precision Killing of M2 Macrophages with Phage-Displayed Peptide-Photosensitizer Conjugates
Source: Cancers (Basel). 2023 Mar 28;15(7):2009. doi: 10.3390/cancers15072009 (PMC10093000; doi:10.3390/cancers15072009)
Supplement: Supplementary file 1 [file cancers-15-02009-s001.zip › cancers-2315887-supplementary.pdf]

Supplementary Materials

Mouldy Sioud and Qindong Zhang

Precision Killing of M2 Macrophages with Phage-displayed peptide-photosensitizer Conjugates

Cancers 2023, 15.

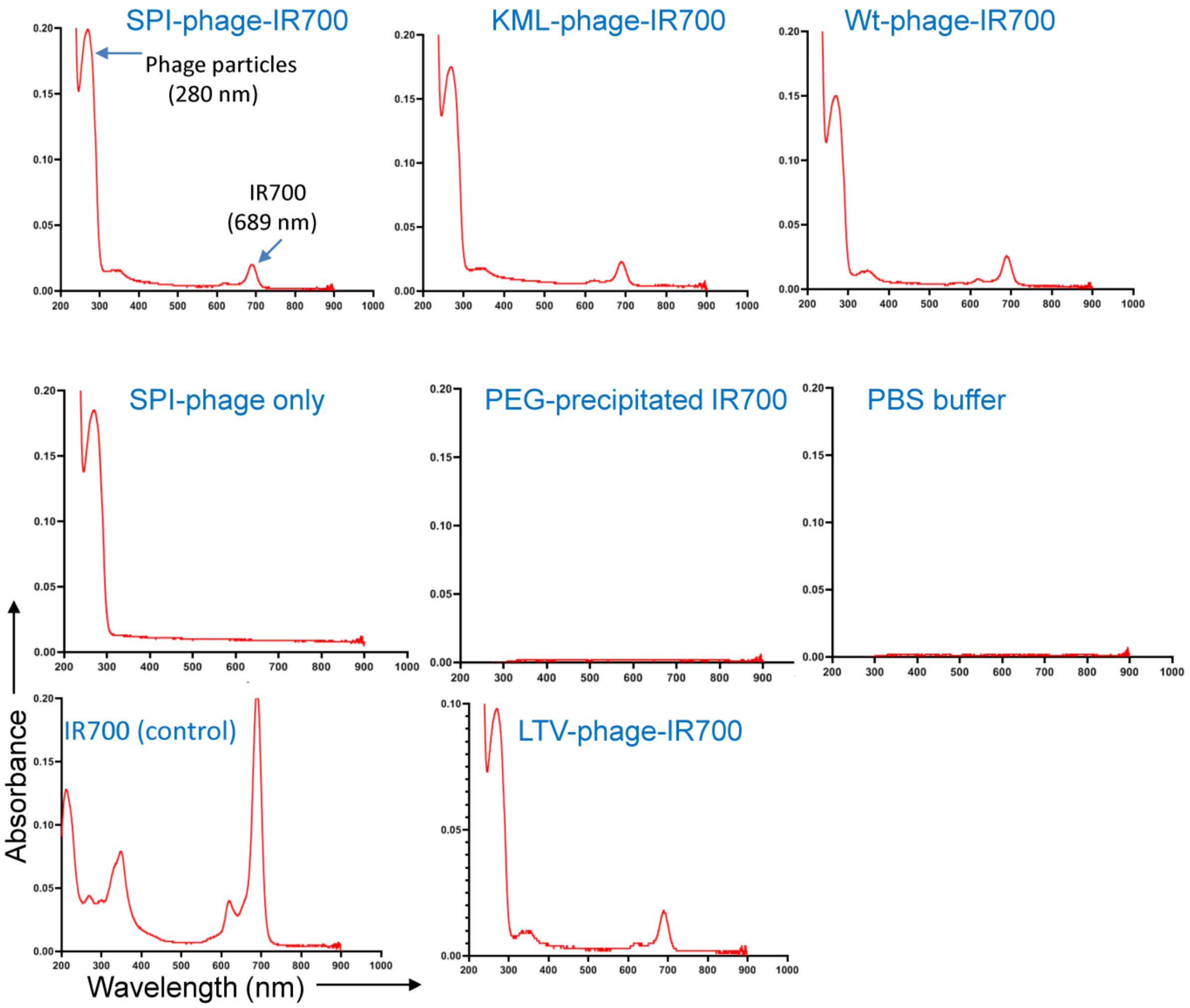

Figure S1. UV-Vis absorbance spectra of the phage-IR700 conjugates after PEG precipitation. UV-Vis absorption spectra were acquired on an Shimadzu spectrophotometer (UV 2550) in 1 ml quartz cell at room temperature in PBS buffer. The spectra were carried out between 200 nm and 900 nm, then values were collected and processed. In contrast to the phage-IR700 conjugates, non-conjugated phages (e.g., SPI phage only) showed no absorption at 689 nm that is specific for the IR700 dye. Of note, free non-conjugated IR700 molecules were not recovered after PEG precipitation (sample PEG-precipitated IR700). Notably, the absorbance spectrum of the sample is comparable to that of the PBS buffer.

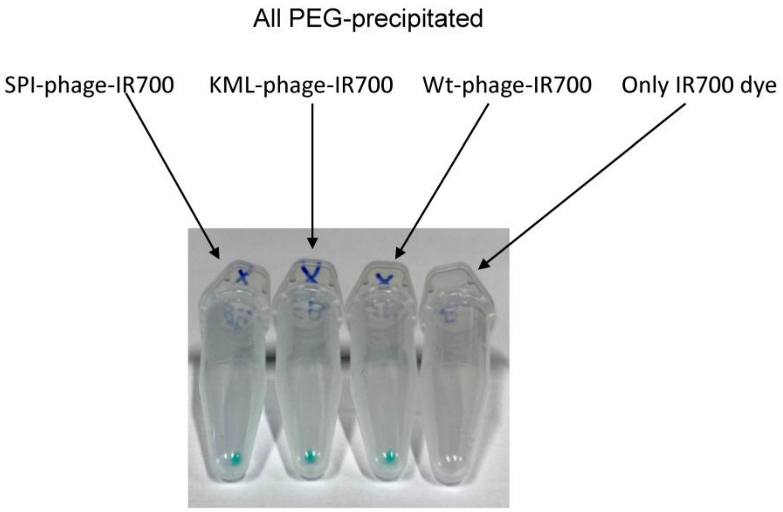

Figure S2. Polyethylene glycol (PEG) precipitation of the conjugated phages

After conjugation, the phages were precipitated as described in Material and Methods. To indirectly investigate whether free non-conjugated IR700 molecules are present in the phage preparations, we added IR700 dye (20 μg) to 1 ml conjugation buffer, quenched the mixture with glycine buffer and then performed PEG precipitation. Under the same conditions used for the phage precipitation, free IR700 molecules were not recovered as shown in the image and further confirmed by spectroscopy analysis (see **Figure S1**).

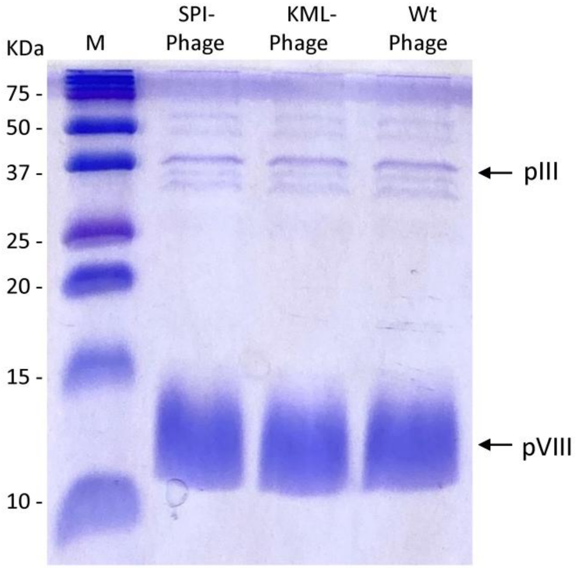

Figure S3. Analysis of the IR700-conjugated phages by 15% SDS-PAGE

An aliquot of each PEG-precipitated phage preparations (around $5x10^8$TU/sample) was mixed 1:1 with sample loading buffer, boiled for 5 min, electrophoresed, and then the gel was coomassie-stained. As expected the major protein band corresponds to the phage coat protein pVIII (around 2700 copies/phage). There was no major difference in electrophoretic mobility between the three phage preparations, suggesting that the number of IR700 molecules conjugated to each phage was comparable. Of note, the molecular weight of pVIII is higher than expected (6 kDa), indicating that it has been conjugated to IR700.

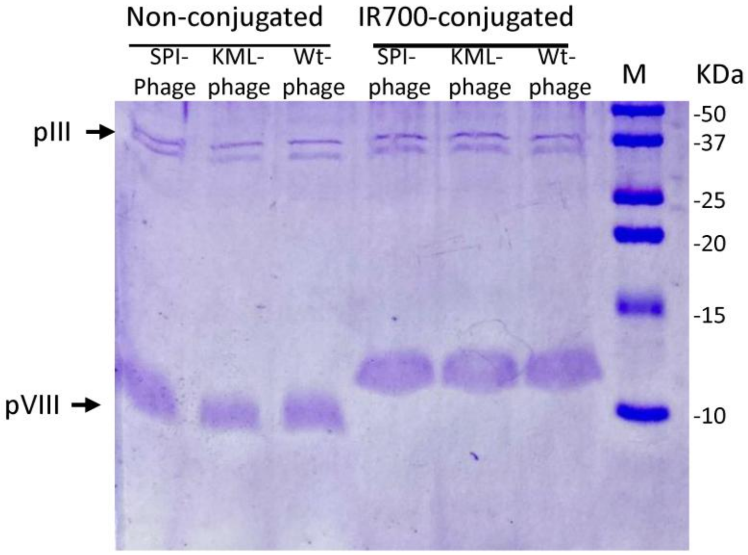

Figure S4. Analysis of non-conjugated and IR700 conjugate phages by 15% SDS-PAGE. Experimental conditions are as **Figure S3**, except that the amount of phages was reduced to $2x10^8$ TU/sample.

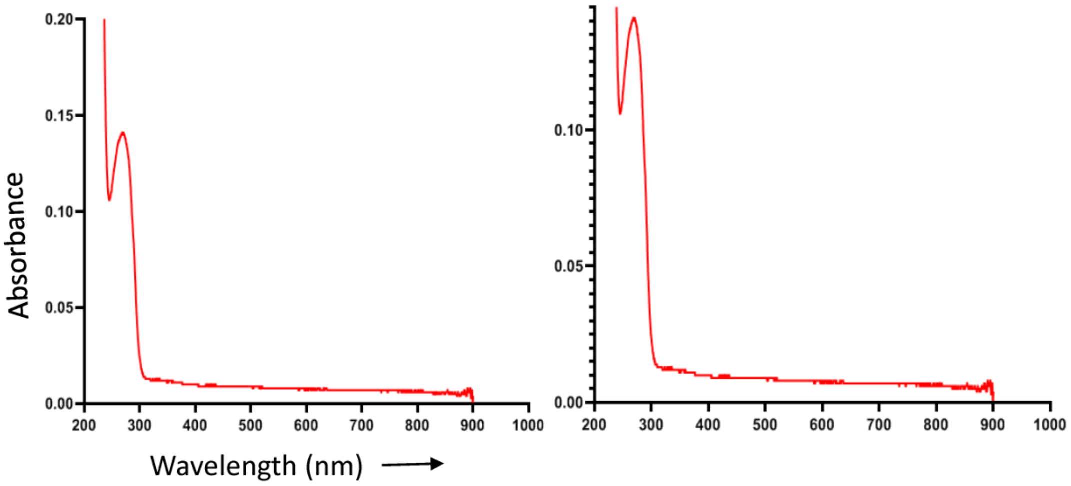

Figure S5. UV-Vis absorbance spectrum. To investigate whether PEG precipitation of phages induces absorption of IR700 molecules on the surface of the phages, the IR700 (20 µg) was added to the phages, the mixture was quenched immediately, and then the phages were precipitated twice as the experimental samples (**Figure S1, see** Materials and Methods). After the first precipitation, the phage pellet was resuspended in 1 ml PBS buffer, vortexed for 30 second and PEG precipitated again. Thereafter, the phage pellet was dissolved in 150 µl PBS and UV-Vis absorption spectrum was acquired as described in **Figure S1**. Notably, no absorption signal was detected at 689 nm.
